# Stem Cell-Derived Exosomes Prevent Aging-Induced Cardiac Dysfunction through a Novel Exosome/lncRNA MALAT1/NF-*κ*B/TNF-*α* Signaling Pathway

**DOI:** 10.1155/2019/9739258

**Published:** 2019-04-08

**Authors:** Bao Zhu, Lulu Zhang, Chun Liang, Bin Liu, Xiangbin Pan, Yanli Wang, Yuqing Zhang, Yu Zhang, Wenping Xie, Bing Yan, Feng Liu, Hon-Kan Yip, Xi-yong Yu, Yangxin Li

**Affiliations:** ^1^Institute for Cardiovascular Science & Department of Cardiovascular Surgery, First Affiliated Hospital of Soochow University, Suzhou, Jiangsu 215123, China; ^2^Department of Cardiology, Shanghai Changzheng Hospital, Second Military Medical University, Shanghai 200003, China; ^3^Department of Cardiology, Second Hospital of Jilin University, Changchun, Jilin 130041, China; ^4^Department of Cardiac Surgery, Fuwai Hospital, Beijing 100037, China; ^5^Division of Cardiology, Department of Internal Medicine, Kaohsiung Chang Gung Memorial Hospital and Chang Gung University College of Medicine, Kaohsiung 83301, Taiwan; ^6^Guangzhou Medical University, Guangzhou, Guangdong 510080, China

## Abstract

Aging is a risk factor for cardiovascular disease, and there is no effective therapeutic approach to alleviate this condition. NF-*κ*B and TNF-*α* have been implicated in the activation of the aging process, but the signaling molecules responsible for the inactivation of NF-*κ*B and TNF-*α* remain unknown. Exosomes have been reported to improve heart functions by releasing miRNA. Recent studies suggest that lncRNAs are more tissue-specific and developmental stage-specific compared to miRNA. However, the role of lncRNA in exosome-mediated cardiac repair has not been explored. In the present study, we focused on metastasis-associated lung adenocarcinoma transcript 1 (MALAT1), which is an lncRNA associated with cell senescence. We discovered that human umbilical cord mesenchymal stem cell- (UMSC-) derived exosomes prevent aging-induced cardiac dysfunction. Silencer RNA against lncRNA MALAT1 blocked the beneficial effects of exosomes. In summary, we discovered that UMSC-derived exosomes prevent aging-induced cardiac dysfunction by releasing novel lncRNA MALAT1, which in turn inhibits the NF-*κ*B/TNF-*α* signaling pathway. These findings will lead to the development of therapies that delay aging and progression of age-related diseases.

## 1. Introduction

Aging is a risk factor for cardiovascular disease [1–4], and oxidative stress has been considered as a possible mechanism underlying aging-related pathologies [5, 6]. It was hypothesized that oxidative stress is associated with inflammation, which is an important contributor of aging. However, the signaling pathway connecting oxidative stress, inflammation, and aging remains undefined, and there is no effective therapeutic approach to alleviate aging-associated cardiovascular disease [4, 7].

Tumor necrosis factor-*α* (TNF-*α*), one of major inflammatory cytokines, is regulated by nuclear factor kappa B (NF-*κ*B). NF-*κ*B is a transcription factor that contains five subunits including p65, RelB, c-Rel, p50, and p52. p65/p50 is retained in the cytosol through its binding to I*κ*B. Upon phosphorylation by IKK, I*κ*B is ubiquitinated and degraded, subsequently allowing p65/p50 to enter the nucleus and bind to the promoter of its target genes. It was reported that ischemic injury triggers the activation of NF-*κ*B, which activates the transcription of inflammatory cytokines such as TNF-*α* [8]. However, whether NF-*κ*B regulates TNF-*α* in the aging process is not known.

It is known that mesenchymal stem cells (MSC) can improve heart function after infarction [9–12], and the beneficial effect of MSCs is mediated by paracrine factors which are transported by exosomes [13]. Exosomes contain functional miRNAs and long noncoding RNAs (lncRNA) and serve as intercellular shuttles to deliver important messages to alter the gene expression and cellular functions of distant organs [14–20]. We and others have reported that bone marrow MSC-derived exosomes improve heart function after infarction, and several miRNA-mediated exosomes' repair functions [12, 21]. However, it is unknown whether exosomes could prevent aging-induced cardiac dysfunction.

Because lncRNAs are more tissue-specific and developmental stage-specific compared to miRNA, we chose to investigate the role of lncRNA in exosomes. More recently, one report showed that lncRNA metastasis-associated lung adenocarcinoma transcript 1 (MALAT1) is associated with the aging process [22]. However, it is unknown whether MSC exosomes contain lncRNA MALAT1 and whether lncRNA MALAT1 in exosomes could have a functional role in preventing aging-induced cardiac dysfunction.

In this study, we explored whether umbilical mesenchymal stem cell- (UMSC-) derived exosomes could prevent aging-induced cardiac dysfunction and determined whether the potential mechanism was mediated by the exosome/lncRNA MALAT1/NF-*κ*B/TNF-*α* pathway.

## 2. Methods

### 2.1. Isolation of Exosomes

Exosomes harvest and identification were performed as reported previously from our lab [21]. Human umbilical mesenchymal stem cells (UMSC) (purchased from Jiangsu Heze Biotechnology Co. Ltd., China) were cultured in DMEM/F12 containing 10% FBS. The FBS has been centrifuged at 100,000 g in order to eliminate preexisting bovine-derived exosomes. After 48 hours of culture, exosomes were isolated from UMSC culture supernatants using total exosome isolation kit (Life Technology, California, USA), which yields high quantities of purified exosomes. The culture media collected from UMSCs were centrifuged at 2,000 g for 30 minutes to remove dead cells and debris, and then the media were transferred to a new tube containing 0.5 volumes of the Total Exosome Isolation reagent. The mixture was incubated at 4°C overnight and centrifuged at 10,000 g for 1 hour at 4°C. The pellets were resuspended in PBS and stored at −80°C. The protein concentration of exosomes was determined using a BCA protein assay kit (Takara, Japan). Exosomes were analyzed by flow cytometry based on the exosome surface maker CD63. Because exosomes are not big enough to be detected directly by flow cytometry, the exosomes were prebound to aldehyde/sulfate latex beads (4 *μ*m; Molecular Probes; Invitrogen) to amplify the channel signal as we have described previously [21]. The gate strategy was based on the diameter of the latex beads (4 *μ*m).

### 2.2. Animal Study

C57BL/6 male mice were obtained from the Experimental Animal Center of Soochow University (Suzhou, China). Animal experiments were approved by the Institutional Animal Care and Use Committee of Soochow University. All of the procedures were in compliance with the Guide for the Care and Use of Laboratory Animals, published by the US National Institutes of Health (NIH Publication number 85-23, revised 1996). D-Galactose (D-gal) was used to induce aging in animals, and the inducing period is relatively shorter compared to the spontaneous aging process [23, 24]. Eight-week C57BL/6 male mice were randomized into the control and aging-induced groups. The control group received normal saline (0.9% NaCl, 0.1 mL/mice). Aging was induced by injection with 200 mg/kg of D-gal (Sigma, Missouri, USA) subcutaneously. The injection was performed every day for 6 weeks. Mice were randomly assigned; exosomes were injected through the tail vein 3 times/week for 6 weeks (100 *μ*g/every time). Mice were either sham-infused with PBS or infused with exosomes. After 6 weeks, mice were anesthetized and tissues were removed and snap-frozen in liquid nitrogen and stored at −80°C until being processed.

### 2.3. Reagents

The antibodies against p-p65, anti-p65, IKK*β*, and goat anti-mouse secondary antibodies were purchased from Cell Signaling Technology (Massachusetts, USA). TNF-*α* and p21 antibodies were purchased from Abcam (Cambridge, UK). Goat anti-rabbit secondary antibody was purchased from Beyotime Biotechnology (Shanghai, China). IKK*β*-specific inhibitor (IMD-0354) was purchased from Selleck (Texas, USA). The siRNA directed against IKK*β* was purchased from RiboBio (Guangzhou, China). Lipofectamine 2000 was purchased from Invitrogen (California, USA). Exosome isolation kit was purchased from Life Technologies (California, USA).

### 2.4. Cell Culture and Treatments

The rat cardiomyocyte cell line H9C2 was purchased from ATCC (Maryland, USA) and cultured in Dulbecco's modified Eagle's medium (DMEM) (Gibco, California, USA), supplemented with 10% fetal bovine serum (FBS), L-glutamine, and 100 *μ*g/mL penicillin/streptomycin. Cells were maintained in a humidified incubator at 37°C with 5% CO_2_. The culture medium was replenished every two days.

When cell populations reached 60–70% confluence, the H9C2 cardiomyocytes were treated with H_2_O_2_ (Lingfeng Chemical Reagent, Shanghai, China) at a final concentration of 200 *μ*M. The cells were harvested for RNA and protein extraction 12 hours after H_2_O_2_ treatment. To study the effects of IMD-0354, siIKK*β*, and exosomes, cells were pretreated with these agents for various times. The final concentrations of these agents are as follows: IMD-0354 (2.5 *μ*M), siIKK*β* (100 nM), and exosomes (200 *μ*g/mL).

### 2.5. Evaluation of Cardiac Function

Echocardiography was performed to evaluate cardiac function at 42 days after exosome injection using a 13 MHz transducer (VisualSonics). The left ventricular ejection fraction (EF) and fraction shorting (FS) were calculated. All procedures and analysis were performed by a researcher who was blinded to treatment groups.

### 2.6. Real-Time PCR

RNA was extracted using TRIzol Reagent (Takara, Japan). Total RNA was reverse-transcribed using PrimeScript RT reagent Kit (Takara, Japan). Real-time PCR was performed using SYBR Premix Ex Taq Kit (Takara, Japan) and the Applied Biosystems 7500 Real-Time PCR System (ABI, CA, USA) with the following primers. H9C2 GAPDH: forward, 5′-CAACGGGAAACCCATCACCAT-3′ and reverse, 5′-AGATGATGACCCTTTTGGCCCC-3′; H9C2 TNF-*α*: forward, 5′-ACTGAACTTCGGGGTGATCG-3′ and reverse, 5′-TGGTGGTTTGCTACGACGTG-3′; H9C2 p21: forward, 5′-GGGATGCATCTATCTTGTGATATGT-3′ and reverse 5′-AGACGACGGCATACTTTGCT-3′; mouse GAPDH: forward, 5′-AAATGGTGAAGGTCGGTGTG-3′ and reverse, 5′-TGAAGGGGTCGTTGATGG-3′; mouse TNF-*α*: forward, 5′-CAGAAAGCATGATCCGCGAC-3′ and reverse, 5′-GGGAACTTCTCATCCCTTTGG-3′; mouse p21: forward, 5′-CCTGGTGATGTCCGACCTG-3′ and reverse, 5′-CCATGAGCGCATCGCAATC-3′; mouse MALAT1: forward, 5′-GGCGGAATTGCTGGTAGTTT-3′ and reverse, 5′-AGCATAGCAGTACACGCCTT-3′; human GAPDH: forward, 5′-GGTGGTCTCCTCTGACTTCAACA-3′ and reverse, 5′-GTTGCTGTAGCCAAATTCGTTGT-3′; human MALAT1: forward, 5′-GACGGAGGTTGAGATGAAGC-3′ and reverse, 5′-ATTCGGGGCTCTGTAGTCCT-3′; human Lethe: forward, 5′-ACAATGAAGCCAAACTGCCG-3′ and reverse, 5′-AGTTTGTCCAAGGGACCCCA-3′; and human H19: forward, 5′-TACAACCACTGCACTACCTG-3′ and reverse, 5′-TGGCCATGAAGATGGAGTCG-3′. GAPDH was used as internal control.

### 2.7. Western Blot Analysis

Cells were washed with PBS and lysed in lysis buffer on ice for 30 minutes. After centrifugation at 12,000 g centrifugation for 10 minutes, the protein content of the supernatant was determined using BCA kit. The protein extracts were separated by polyacrylamide gel electrophoresis (12–15%) and transferred to polyvinylidene difluoride (PVDF) membranes. The membranes were probed with primary antibodies against TNF-*α*, p-p65, p65, and p21, followed by a goat anti-rabbit IgG–horseradish peroxidase antibody. All proteins were visualized by ECL Chemiluminescent Kit (Biological Industries, Israel), and chemical luminescence was detected by the Bio-Rad luminescent imaging system.

### 2.8. Small Interference RNA (siRNA) Synthesis and Treatment

An siRNA directed against IKK*β* was designed to target the coding region of the IKK*β* mRNA (position 507-525, 5-TCTTAGATACCTTCATGAA-3). Scrambled siRNAs which do not lead to the specific degradation of any known cellular mRNA were used as negative control. Before the start of the experiment, 3 × 10^5^ H9C2 cells were inoculated in 6-well plates. After the cell density reached 50% to 60% confluence, the siRNAs (100 nM) were delivered into cultured H9C2 cells by Lipofectamine 2000 from Invitrogen (California, USA) following the manufacturer's protocol. RNAs were extracted 24 h after transfection for real-time RT-PCR analyses, and cell lysates were prepared 48 h after transfection for Western blot.

MALAT1 was knocked down using the Ribo™ lncRNA Silencer, which was a pool of siRNAs: 5-AAGGTCAAGAGAAGTGTCA-3; 5-TCCCTCTTTCTGGAGTGAA-3; 5-TGGAGAAGCTCTAAATTGT-3; 5-AAGTAGGACAACCATGGAGC-3; 5-TTGATCTAGCACAGACCCTT-3; and 5-GAGAAGCCCTACTGCTGAAA-3. Scrambled siRNAs were used as negative control. When the cell density of UMSC reached 50% to 60% confluence, the siRNAs (100 nM) were transfected into the cells by Lipofectamine 2000 from Invitrogen (California, USA) following the manufacturer's protocol. Exosomes were harvested 48 h after transfection as reported previously from our lab [21].

### 2.9. Dual-Luciferase Reporter (DLR) Assay

Rat cardiomyocyte H9C2 cells were seeded on 24-well plates in DMEM containing 10% FBS, then cotransfected with 500 ng NF-*κ*B-Luc and 50 ng PRL-TK plasmid (internal control) using Lipofectamine 2000. Forty-eight hours post-transfection, cells were either treated with H_2_O_2_ (200 *μ*M) for 6 h or pretreated with IMD-0354 for 1 h, or exosomes for 24 h, followed by H_2_O_2_ treatment, and then DLR assays were performed using a luciferase assay kit (Promega, WI, USA).

### 2.10. TNF-*α* ELISA Assay

The levels of TNF-*α* mouse serum were determined using an ELISA Kit per manufacturer's instructions (Abcam, UK).

### 2.11. Exosome Labeling with PKH26

Purified exosomes were labeled using a PKH26 fluorescent labeling kit (Sigma-Aldrich, USA), as described in our previous publication [21].

### 2.12. Senescence-Associated *β*-Galactosidase (SA-*β*-gal) Staining

Cells were seeded onto 96 wells and cultured until reaching 70% confluence. SA-*β*-gal staining was performed using the Senescence *β*-Galactosidase Staining Kit (Beyotime, Shanghai, China). The cells were incubated 24 h with 1x SA-*β*-gal detection solution prepared as described in the protocol at 37°C without CO_2_ and protected from light. Cells were quantified in three different fields under a light microscope.

### 2.13. EdU Proliferation Assay

The effects of exosomes on H9C2 cell proliferation were determined using the EdU Cell Proliferation Assay Kit (RiboBio, Guangzhou, China). H9C2 cells were seeded in 96-well plates and pretreated with exosomes (200 *μ*g/mL) for 24 hours followed by 200 *μ*M H_2_O_2_ for 3 hours. The cells were incubated with either 50 *μ*M EdU for 2 h (in complete medium with serum) or 10 *μ*M EdU for 24 h (in serum-free medium) before fixation and permeabilization. The cell nuclei were stained with DAPI (Sigma, USA) at a concentration of 200 ng/mL for 15 minutes. The proportion of the cells incorporating EdU was determined with fluorescence microscopy.

### 2.14. Statistical Analysis

Data were presented as mean ± SEM. Statistical analysis was performed by using Student's *t* test when appropriate. *p* < 0.05 was considered significant. All experiments were performed at least 3 times.

## 3. Results

### 3.1. Exosomes Prevent the Aging Process Mediated by lncRNA MALAT1

**Figure 1:**
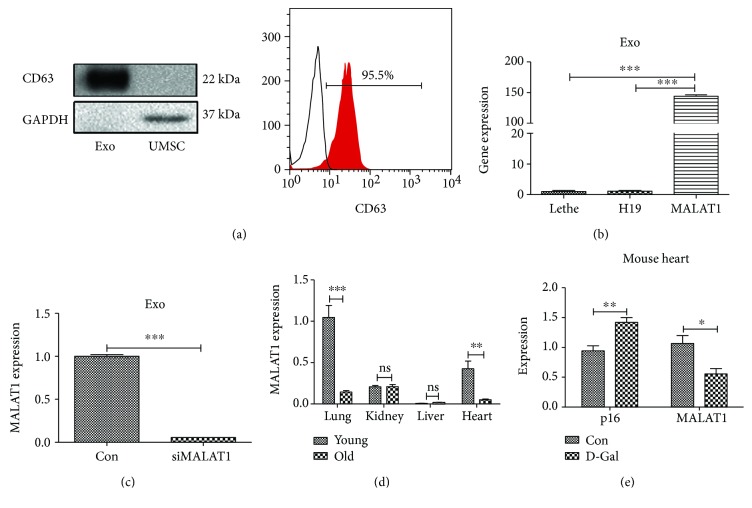
The expression of lncRNA MALAT1 is tissue- and age-specific. (a) Levels of the exosomal surface marker CD63 was determined by Western blot and flow cytometry. (b) Total RNA from exosomes was extracted, and the levels of MALAT1, H19, and Lethe were measured using qRT-PCR. (c) siMALAT1 downregulated the expression of lncRNA MALAT1 in exosomes. (d) The expression of MALAT1 in young and aged hearts was analyzed by qRT-PCR (*N* = 4/group). (e) D-Gal treatment decreased the expression of MALAT1 and increased the expression of p16 in the heart (*N* = 4/group). ns: nonsignificant. ^∗^*p* < 0.05, ^∗∗^*p* < 0.01, and ^∗∗∗^*p* < 0.001.

To determine whether exosomes could prevent the aging process and whether the effect was mediated by lncRNA, exosomes were isolated from UMSCs as described in Methods and our previous publication. The expression of the exosome marker CD63 was analyzed by western blot and flow cytometry (Figure 1(a)). We found that the expression of the novel potential aging regulator lncRNA MALAT1 was much higher than those of other lncRNAs such as H19 and Lethe in exosomes (Figure 1(b)). The siRNA targeting lncRNA MALAT1 (siMALAT1) was able to downregulate the expression of lncRNA MALAT1 in exosomes (Figure 1(c)). Moreover, the expression of lncRNA MALAT1 was decreased in aging hearts (Figure 1(d)) and in hearts treated with D-gal which has been used as an aging-inducing agent, while the expression of p16 was increased in the hearts treated with D-gal (Figure 1(e)).

**Figure 2:**
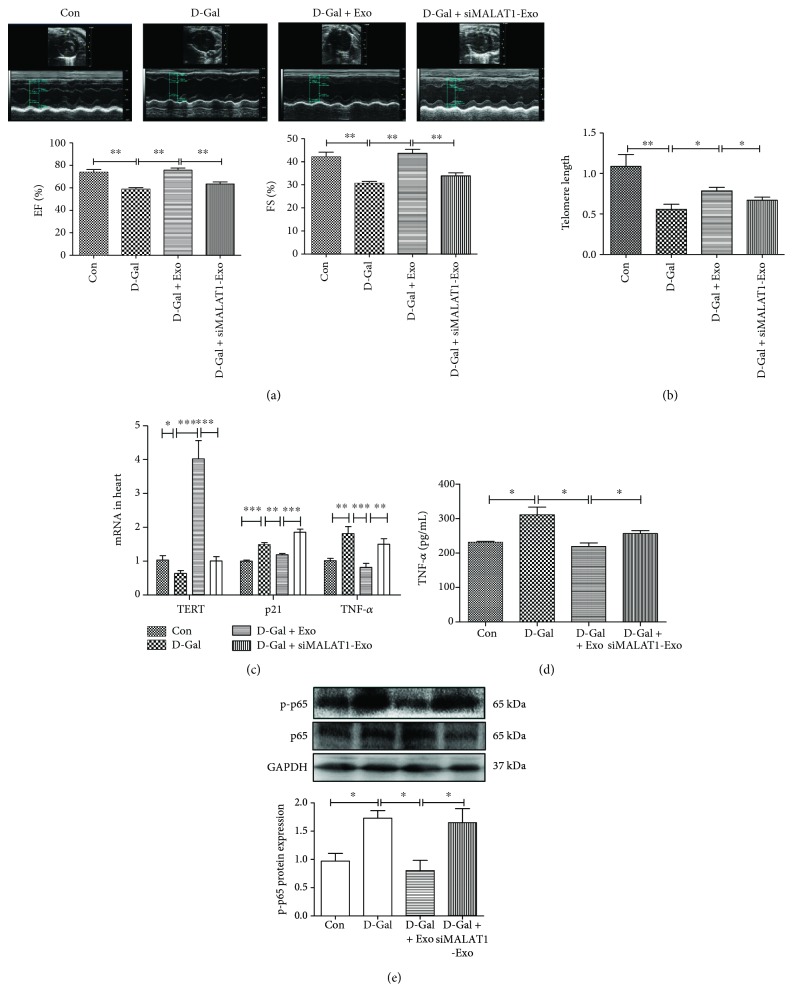
Exosomes prevent the aging process through the lncRNA MALAT1 signaling pathway. (a) Representative echocardiography images of left ventricular ejection fraction (EF) and fraction shorting (FS) in control, D-gal-, D-gal + EXO- and D-gal + siMALAT1-EXO-treated groups (*n* = 4/group). (b) The telomere length in control, D-gal, D-gal + EXO, and D-gal + siMALAT1-EXO mouse heart tissue (*n* = 4/group). (c) The effects of D-gal, D-gal + EXO, and D-gal + siMALAT1-EXO on the expression of TERT, p21, and TNF-*α* mRNA levels in heart (*n* = 4/group). (d) The effects of D-gal, D-gal + EXO, and D-gal + siMALAT1-EXO on circulating levels of TNF-*α*. (e) EXO reduced cardiac levels of NF-*κ*B component p-p65. *n* = 4/group. ^∗^*p* < 0.05, ^∗∗^*p* < 0.01, and ^∗∗∗^*p* < 0.001.

To determine whether exosomes could prevent aging-induced cardiac dysfunction, exosomes were injected into D-gal-treated mice. We found that exosomes attenuated the effects of D-gal in the left ventricular ejection fraction (EF) and fraction shorting (FS), and siMALAT1 blocked the function of exosomes (Figure 2(a)). We also found that exosomes attenuated the effects of D-galactose (D-gal) on telomere length, and the beneficial effects of exosomes were blocked by siMALAT1 (Figure 2(b)).

To explore the potential mechanism, we examined the impact of injected exosomes on the expression of aging and inflammatory factors in the heart. RT-PCR analysis showed that antiaging marker TERT was increased, while the aging marker p21 and inflammatory marker TNF-*α* was decreased in exosome-treated mice (Figure 2(c)). The circulating levels of TNF-*α* was also decreased in the exosome-treated group (Figure 2(d)). Western blot analysis revealed that cardiac NF-*κ*B component p-p65 was decreased in the exosome-treated group (Figure 2(e)). These protective effects of exosomes were blocked by siMALAT1. These data suggest that the antiaging effects exerted by exosomes are mediated by lncRNA MALAT1 and subsequent inhibition of the NF-*κ*B/TNF-*α* pathway.

### 3.2. NF-*κ*B Activity Is Increased in an In Vitro Aging Model

**Figure 3:**
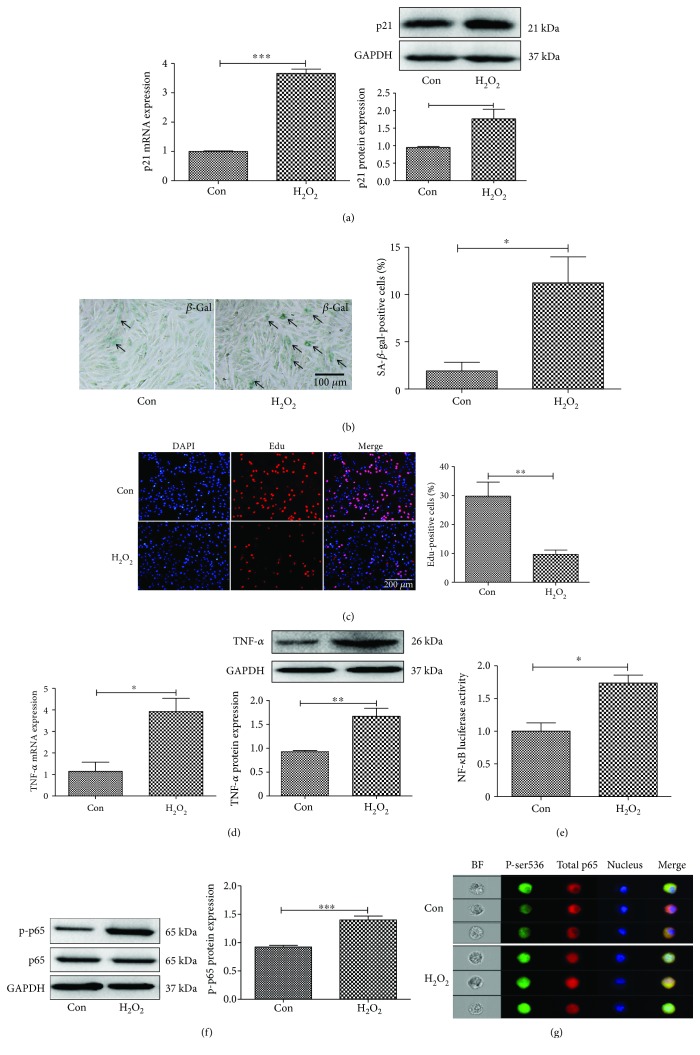
H_2_O_2_ induces senescence in cardiomyocytes. (a) The mRNA and protein expression levels of p21 was increased in H9C2 cells treated with 200 *μ*M H_2_O_2_ for 12 h. (b) Immunohistochemistry staining of *β*-gal in cardiomyocytes treated with H_2_O_2_. (c) Edu staining for cell proliferation. (d) TNF-*α* mRNA levels were determined by real-time PCR, and TNF-*α* protein levels were analyzed by Western blot. (e) Dual luciferase assay revealed increased NF-*κ*B activity in H_2_O_2_-treated cells. (f) Western blot and (g) flow imaging revealed increased expression of p-p65 in H_2_O_2_-treated cells. *N* = 3/group. ^∗∗^*p* < 0.01 and ^∗∗∗^*p* < 0.001.

An in vitro aging model was set up to explore the potential mechanism. The aging process is associated with cell senescence, and previous studies showed that oxidative stress plays important roles in cell senescence and the aging process [25]. H_2_O_2_, the main component of reactive oxygen species (ROS), is an inducer of oxidative stress. Thus, H_2_O_2_ (200 *μ*M) was used to treat cardiomyocytes H9C2 as the *in vitro* aging model. As shown in Figure 3(a), the mRNA and protein expression levels of p21 were increased in H9C2 after the cells were treated with 200 *μ*M H_2_O_2_ for 12 h (Figure 3(a)). The increase of *β*-gal-positive cells by H_2_O_2_ treatment confirmed that the in vitro aging model has been successfully established (Figure 3(b)). The reduced cell proliferation was consistent with the aging process (Figure 3(c)). The mRNA and protein expression levels of inflammatory factor TNF-*α* were increased in the H_2_O_2_-treated group compared to control (Figure 3(d)). H_2_O_2_ treatment enhanced NF-*κ*B activity (Figure 3(e)). The expression levels of NF-*κ*B key subunit p-p65 were also increased as determined by western blot and flow imaging (Figures 3(f) and 3(g)).

### 3.3. Cell Senescence Is Mediated by the NF-*κ*B Signaling Pathway

**Figure 4:**
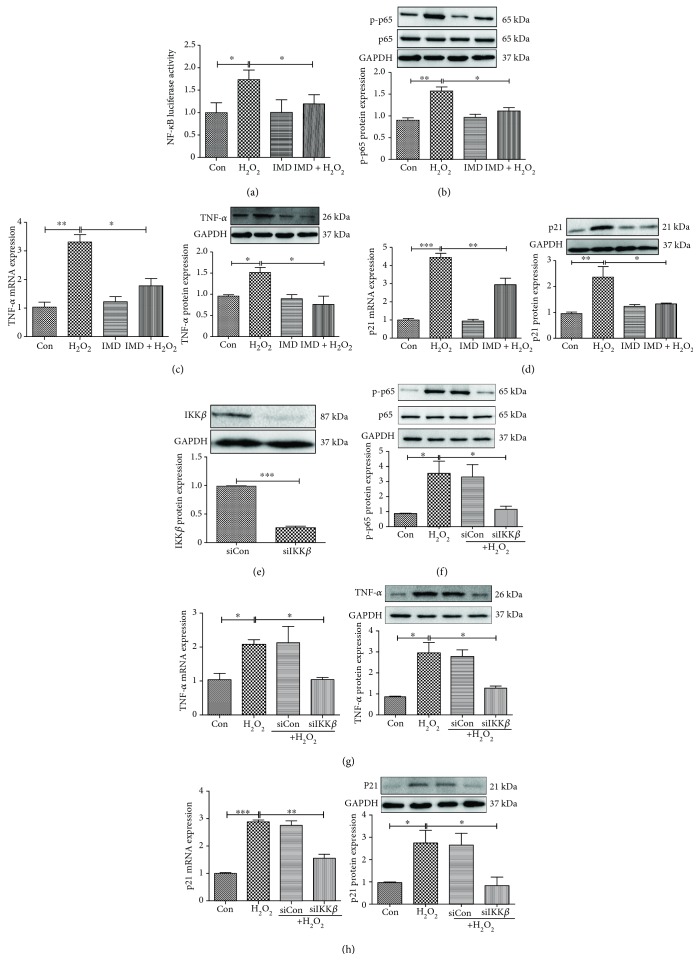
Inactivation of the NF-*κ*B signaling pathway prevents H_2_O_2_-induced cell senescence. (a) Dual luciferase assay revealed increased NF-*κ*B activity in H_2_O_2_-treated cells, and IMD-0354 (IMD), a specific IKK*β* inhibitor, inhibited H_2_O_2_-induced NF-*κ*B activation. (b) IMD inhibited H_2_O_2_-induced expression of p-p65 in cardiomyocytes. (c) IMD inhibited H_2_O_2_-induced TNF-*α* mRNA and protein expression. (d) IMD inhibited H_2_O_2_-induced p21 mRNA and protein expression. (e) Transfection of cardiomyocytes with siRNA targeting IKK*β* (siIKK*β*) resulted in reduction of IKK*β* protein expression. (f) IKK*β* silencing in cardiomyocytes led to reduced expression of p-p65. (g) IKK*β* silencing in cardiomyocytes led to reduced expression of TNF-*α*. (h) IKK*β* silencing in cardiomyocytes led to reduced expression of p21. *N* = 4/group, ^∗^*p* < 0.05, ^∗∗^*p* < 0.01, and ^∗∗∗^*p* < 0.001.

NF-*κ*B is regulated by IKK*β* [26, 27]. To determine whether NF-*κ*B signaling mediated inflammation and the cell senescence process, we examined the effect of IMD-0354 (IMD), a specific IKK*β* inhibitor, in an *in vitro* aging model. H9C2 cardiomyocytes were transfected with NF-*κ*B Luc and RL-TK by Lip 2000 for 48 h, then treated with H_2_O_2_ for 12 h. H_2_O_2_ treatment enhanced NF-*κ*B activity (Figure 4(a)). IMD inhibited NF-*κ*B activity (Figure 4(a)) and decreased the expression of NF-*κ*B subunit p-p65 in H_2_O_2_-treated cardiomyocytes (Figure 4(b)). As a result, IMD inhibited H_2_O_2_-induced inflammation (Figure 4(c)). Moreover, IMD inhibited the expression of p21, an important marker of cell senescence (Figure 4(d)).

To confirm these findings, we silenced IKK*β*, the upstream kinase that regulates the activation of NF-*κ*B. As shown in Figure 4(e), transfection of cardiomyocytes with siRNA targeting IKK*β* (siIKK*β*) resulted in reduction of IKK*β* protein expression. Similar to what we found with NF-*κ*B inhibitor IMD, IKK*β* silencing in cardiomyocytes led to reduced expression of p-p65 (Figure 4(f)) and TNF-*α* in the H_2_O_2_ treatment group (Figure 4(g)). Transfection of siIKK*β* also inhibited H_2_O_2_-induced p21 expression (Figure 4(h)).

### 3.4. Exosomes Inhibit the NF-*κ*B/TNF-*α* Signaling Pathway

**Figure 5:**
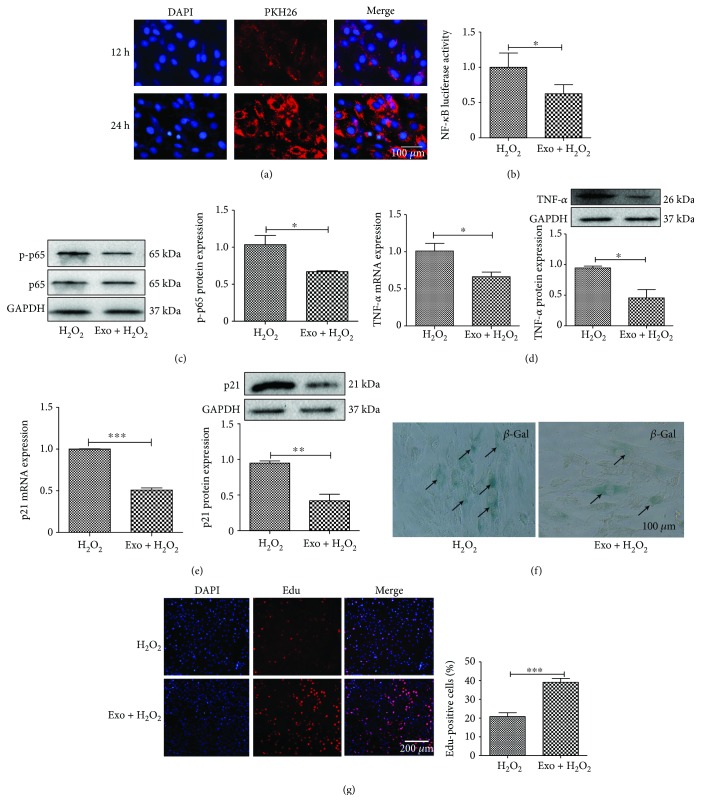
EXO inhibits the NF-*κ*B signaling pathway. (a) H9C2 was incubated with PKH26-labeled exosome (EXO), and the uptake of EXO was observed under a fluorescent microscope. (b) Luciferase assay for NF-*κ*B activation. Cardiomyocytes were transfected with NF-*κ*B-luc and RL-TK plasmids by Lip 2000 for 24 h, and EXO (200 *μ*g/mL) was added to the cells and incubated for another 24 h. The cells were then treated with H_2_O_2_ for 6 h. (c) Western blot analysis of p-p65 expression. (d) RT-PCR and Western blot analysis of TNF-*α* expression. (e) RT-PCR and Western blot analysis of p21 expression. (f) Immunohistochemistry staining of *β*-gal in cardiomyocytes treated with EXO. (g) Edu staining for cell proliferation treated with EXO. *N* = 4/group^∗^*p* < 0.05, ^∗∗^*p* < 0.01, and ^∗∗∗^*p* < 0.001.

To define the mechanisms underlying exosome-mediated inhibition of the aging process, we incubated the exosomes with cardiomyocytes and assessed the effect of exosomes on H_2_O_2_-induced inflammation and senescence. As shown in Figure 5(a), a significant amount of exosomes entered the cells after coincubation with the cells for 24 hours (Figure 5(a)). We further demonstrated that exosomes inhibited H_2_O_2_-induced NF-*κ*B activity (Figure 5(b)) and the expression of NF-*κ*B subunit p-p65 (Figure 5(c)). Exosomes also inhibited H_2_O_2_-induced expression of TNF-*α* at both mRNA and protein levels (Figure 5(d)).

We also assessed the effect of exosomes on cardiomyocyte senescence. RT-PCR and Western blot analysis showed that exosome pretreatment prevented H_2_O_2_-induced expression of senescence gene p21 at both mRNA and protein levels (Figure 5(e)). Exosomes also prevented H_2_O_2_-induced *β*-gal-activity (Figure 5(f)). Edu staining revealed that exosomes promoted cardiomyocyte proliferation (Figure 5(g)).

### 3.5. Exosomes Prevent Cell Senescence through the lncRNA MALAT1 Signaling Pathway

**Figure 6:**
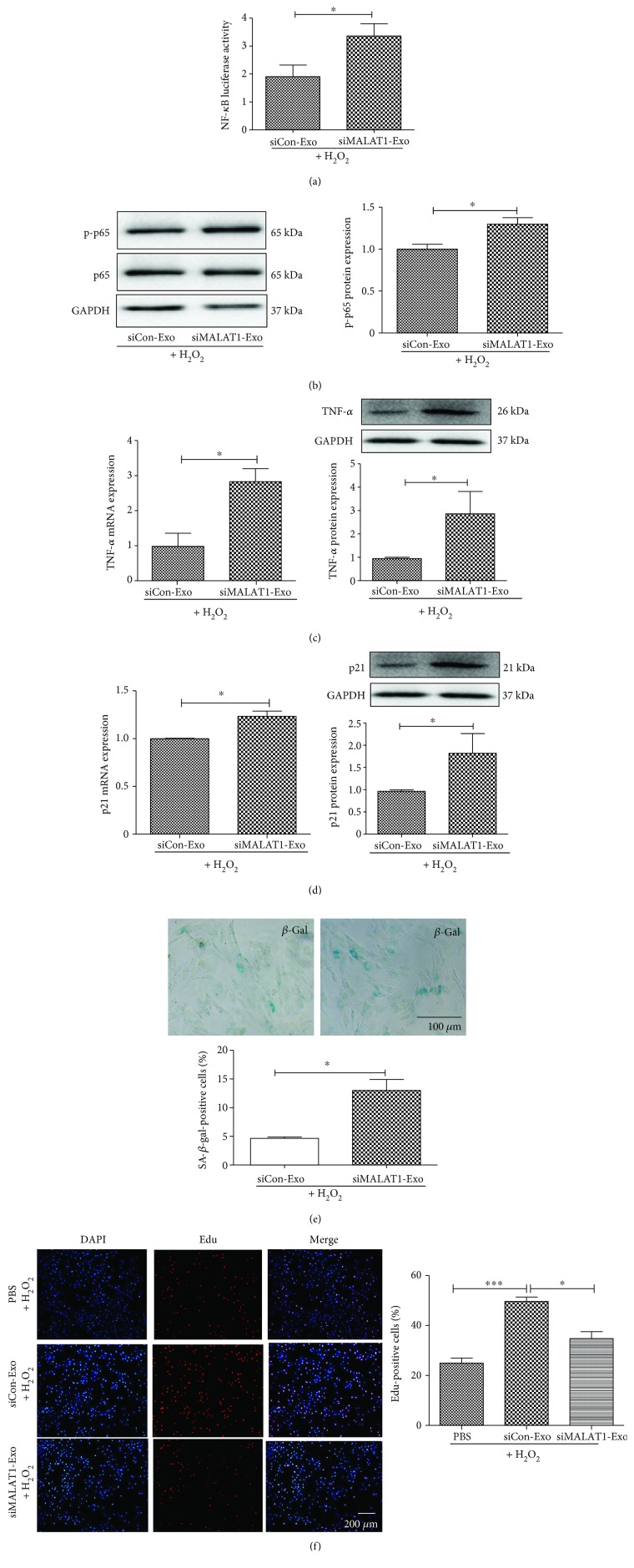
EXO prevents cell senescence through the regulation of lncRNA MALAT1. (a) Dual luciferase assay revealed siRNA-MALAT1 reversed the EXO's effects on NF-*κ*B activity in H_2_O_2_-treated cells. (b) siRNA-MALAT1 blocked EXO's effect on p-p65 expression. (c) siRNA-MALAT1 blocked EXO's effect on TNF-*α* expression. (d) siRNA-MALAT1 blocked EXO's effect on p21 expression. (e) Immunohistochemistry staining of *β*-gal in cardiomyocytes treated with siRNA-MALAT1. (f) Edu staining for cell proliferation treated with siRNA-MALAT1. *N* = 4/group, ^∗^*p* < 0.05, ^∗∗^*p* < 0.01, and ^∗∗∗^*p* < 0.001.

To further determine whether exosomes could prevent cell senescence through the lncRNA MALAT1 signaling pathway, siMALAT1 was employed in the *in vitro* aging model. As shown in Figure 6, transfection of cardiomyocytes with siMALAT1 led to a reduction in NF-*κ*B activity (Figure 6(a)) and the expression level of NF-*κ*B subunit p-p65 (Figure 6(b)). siMALAT1 also reduced the expression of TNF-*α* induced by H_2_O_2_ treatment (Figure 6(c)). Moreover, transfection of siMALAT1 inhibited H_2_O_2_-induced p21 expression (Figure 6(d)). siMALAT1 blocked the effect of exosomes on *β*-gal-activity (Figure 6(e)) and cell proliferation (Figure 6(f)). These data suggest that exosomes prevent cell senescence through the lncRNA MALAT1/NF-*κ*B/TNF-*α* pathway.

## 4. Discussion

**Figure 7:**
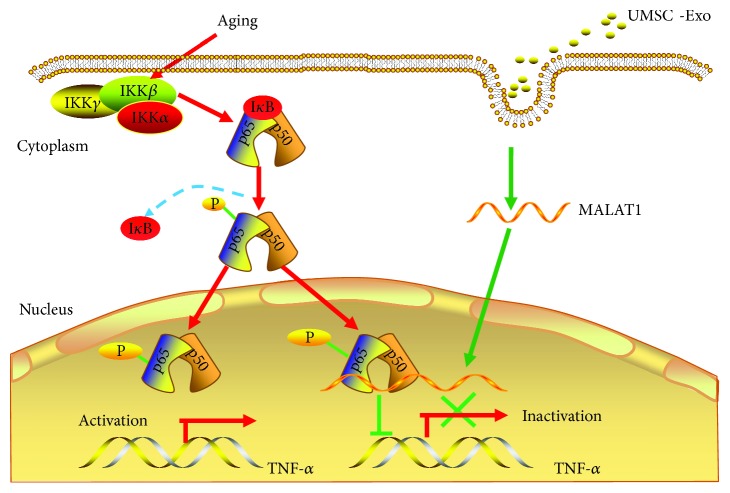
The diagram of mechanisms. The protective effects exerted by exosomes against aging-induced cardiac dysfunction are mediated through the novel exosome/lncRNA MALAT1/NF-*κ*B/TNF-*α* signaling pathway.

In the present study, we discovered that UMSC-derived exosomes prevent aging-induced cardiac dysfunction and the beneficial effects were mediated by the novel exosome/lncRNA MALAT1/NF-*κ*B/TNF-*α* pathway (Figure 7). To our knowledge, this is the first study to use exosomes to prevent aging-induced cardiac dysfunction. Together, this study identified a novel lncRNA signaling pathway to prevent the aging process. UMSC is easy to obtain; thus, exosome-mediated therapy can be used for clinical application.

The aging process is a major risk factor for cardiovascular disease, yet the cellular mechanisms for aging are complex and undefined. Several studies demonstrated that exosomes improve heart function in myocardial infarction and hypertrophy, and the effects were mediated by miRNA. We are the first to show that the beneficial effects of exosomes are mediated by lncRNA MALAT1. The loss of the function approach by silent lncRNA MALAT1 demonstrates that the function of exosomes is mediated by the lncRNA MALAT1/NF-*κ*B/TNF-*α* pathway. This finding not only supports the previous reports that lncRNA MALAT1 regulate cell cycle and inflammation [22, 28] but also for the first time demonstrated that exosomes can be used to deliver lncRNA MALAT1 to alter NF-*κ*B signaling in vivo, which has clinical implications.

It was reported that lncRNA Lethe can downregulate the NF-*κ*B signaling pathway, and its expression is decreased with age [29]. lncRNA H19 prevents apoptosis and stimulates muscle generation [30, 31]. Although Lethe and H19 have a potential role in mediating exosomes' effect during the aging process, they were not detected in exosomes derived from UMSC. Our results showed that among potential antiaging lncRNAs, only lncRNA MALAT1 was highly expressed in exosomes. These results support the notion that lncRNAs are cell-, tissue-, and stage-specific [32, 33]. These characteristics of lncRNA make it a good candidate as a therapeutic agent. Moreover, lncRNA can be protected from RNase degradation when transported by exosomes. That is also the reason that we choose exosomes to deliver lncRNA MALAT1 to prevent the aging process. Furthermore, as a “multisignaling device,” exosomes can deliver signaling molecules to the cytosol and the nucleus. Our results showed that lncRNA MALAT1 was transported to the nucleus to regulate the expression of TNF-*α*.

Although it has been reported that D-gal can be used to induce cardiac aging [34], this model does not reflect all the characteristics of the natural aging process. Our results have proved that MALAT1 can delay the aging process in the D-gal-induced animal model; it would be interesting to investigate the function of MALAT1 in cardiac aging using a natural aging model in the future. Moreover, cell senescence can be induced by several methods including H_2_O_2_ [35], D-gal [36], cell cycle inhibition [37], and radiation mutation [38]. Our present study showed that MALAT1 can delay cell senescence induced by H_2_O_2_, but whether MALAT1 can delay cell senescence in other models remains unknown.

NF-*κ*B is a transcription factor that regulates the expression of several inflammatory mediators such as TNF-*α*, IL-6, and IL-1*β*. It has been shown that knockdown of MALAT1 can downregulate the mRNA expression levels of TNF-*α*, but not IL-6 and IL-1*β* in THP-1 cells [28]. We demonstrated that exosome-derived MALAT1 inhibited TNF-*α* expression in H9C2 cells, but whether MALAT1 can influence the expression of IL-6 and IL-1*β* remains to be determined. Furthermore, it has been reported that cardiac aging is associated with changes in telomere length [4], which can be regulated by lncRNA [39]. Our vivo experiment showed that MALAT1 alters telomere length and telomere reverse transcriptase activity; however, the underlying mechanisms remain to be clarified in future studies.

## 5. Conclusion

In this study, we discovered that UMSC-derived exosomes can prevent aging-induced cardiac dysfunction by regulating the novel exosome/lncRNA MALAT1/NF-*κ*B/TNF-*α* pathway. The development of therapies that delay aging and progression of age-related diseases will have major implications for the improvement of public health.

## Data Availability

The data used to support the findings of this study are available from the corresponding author upon request.
